# Potential Novel Genotype of “Bopivirus B” from Sheep in Türkiye: Epidemiology and Molecular Characterization

**DOI:** 10.3390/pathogens15010052

**Published:** 2026-01-05

**Authors:** Feray Alkan, İlke Karayel-Hacıoğlu, Selda Duran-Yelken, Fruzsina Tóth, Buket Pekşen, Ákos Boros

**Affiliations:** 1 Department of Virology, Faculty of Veterinary Medicine, Ankara University, Ankara 06070, Türkiye; falkan@ankara.edu.tr (F.A.); ilkeekarayel@gmail.com (İ.K.-H.); 2 Department of Virology, Graduate School of Health Sciences, Ankara University, Ankara 06070, Türkiye; buketguldvm@gmail.com; 3 Department of Virology, Faculty of Veterinary Medicine, Kastamonu University, Kastamonu 37150, Türkiye; syelken@kastamonu.edu.tr; 4 Department of Medical Microbiology, Medical School, University of Pécs, 7624 Pécs, Hungary; toth.fruzsina1@edu.pte.hu

**Keywords:** Bopivirus, picornavirus, sheep, phylogenetics, Türkiye

## Abstract

Various microbial agents have been found in the feces of both humans and animals, especially in newborns. While some of these agents are recognized as causing diarrhea, the role of others, specifically bopiviruses of the family *Picornaviridae*, in diarrhea remains uncertain. In this study, we conducted an analysis of 214 fecal samples from cattle (n = 114), sheep (n = 82), and goats (n = 18) with diarrhea, collected from farms across 17 different provinces in Türkiye. All samples were tested using RT-PCR targeting the 3D^(RdRp)^ region of bopiviruses, and two samples from sheep (2.4%) tested positive. The 7303 nt-long complete coding sequence of Bopivirus/Sheep/KS-1M/2024/TUR and partial 3D^(RdRp)^, VP3, and 2A-2C sequences of Bopivirus/Sheep/ANK-K30/2017/TUR were determined by additional RT-PCR, 3′RACE-PCR reactions and Sanger sequencing. Both strains show close sequence and phylogenetic relationship to members of species “Bopivirus B” of genus *Bopivirus*. Bopivirus/Sheep/KS-1M/2024/TUR is most closely related to a sheep Bopivirus B strain (sheep/14-73/2018/ITA) from Italy, but the phylogenetic separation, the low sequence identities and high p-distance values in VP1 to existing genotypes of “B1” and “B2” suggest that both strains could belong to novel genotypes (“B3” and “B4”) in species “Bopivirus B”, although additional closely related sequences are necessary for proper typing.

## 1. Introduction

The family *Picornaviridae* encompasses a large number of genera [1]. According to the International Committee on Taxonomy of Viruses (ICTV), this family currently includes 68 genera and 159 species [2]. Members of the genus *Bopivirus* have c.a. 7.8 kb-long positive-sense single-stranded RNA genomes which contain a single open reading frame (ORF) flanked by 5′ and 3′ untranslated regions (UTRs) and a 3′ terminal poly(A)-tail [3,4]. The 5′UTRs contain a type II internal ribosomal entry site (IRES) [3]. The ORF is organized as follows: VP4-VP2-VP3-VP1-2A-2C-3A-3D [3,4].

Currently, there is only a single officially accepted species (*Bopivirus abovi*, formerly called as *Bopivirus A*) in the genus *Bopivirus*, although the viruses temporarily named “ovipivirus” (from sheep) and “gopivirus” (from goats) in fecal samples collected from clinically healthy animals in Hungarian farms also belonged to this genus [3]. These viruses, which might belong to two genotypes (“B1” and “B2”), show only a distant genetic relationship to bovine bopiviruses of the species *Bopivirus abovi* and, based on ICTV classification criteria, have been proposed to represent a novel species, “Bopivirus B”. Around the same time, a genetically distinct bopivirus (with 57–79% nucleotide similarity to previously reported bovine, ovine, and caprine bopiviruses) was identified in fecal samples from fallow deer and red deer in Australia. This novel virus was also proposed as a member of an additional novel species, “Bopivirus C” [4]. These findings reveal the presence of genetically highly diverse bopiviruses in both domestic and wild even-toed ungulates. In addition to reports from Hungary [3] and Australia [4], relevant studies involving domestic and wild animals have also been reported in Italy [5,6], USA (PV TCH6/2013/USA; GenBank accession no. KM589358, unpublished sequence), China [7,8], and New Zealand [9]. Although bopiviruses have been reported in various species, the virus–host relationship and geographic patterns of bopiviruses have not been fully elucidated. Therefore, studies on molecular characterization of new bopivirus strains from different hosts will provide valuable information about the origin, distribution, and diversity of these viruses.

This study aims to investigate the prevalence and genetic divergence of bopiviruses in sheep, goats, and cattle in Türkiye. By analyzing diagnostic enteric samples obtained from both healthy and diarrheic animals, this study also suggests the potential role of bopiviruses in diarrheal diseases and data on the species and bopivirus genetics.

## 2. Materials and Methods

### 2.1. Diagnostic Samples

**Figure 1:**
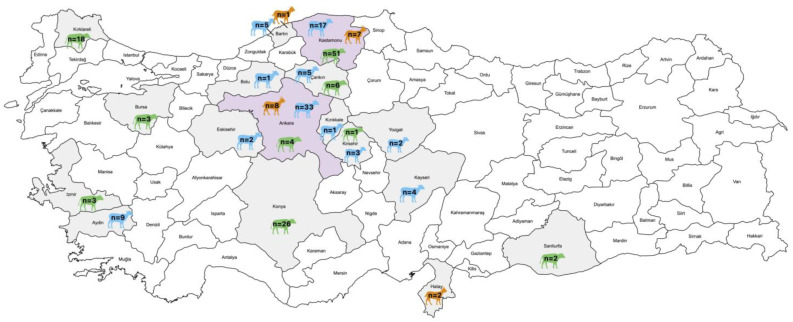
Distribution of collected fecal samples (n = 214) from various provinces, indicating the species and quantity of samples using different colors (The map was constructed using MapChart https://www.mapchart.net/).

This study analyzed 214 fecal samples from three ruminant species: cattle (n = 114), sheep (n = 82), and goats (n = 18). Samples were collected from diarrheic calves under six months of age, as well as from sheep and goats younger than two years, between 2009 and 2024 across 17 provinces. Among the sheep, the samples were obtained from diarrheic animals (n = 63) and clinically healthy animals (n = 19). For the goats, samples were collected from diarrheic (n = 12) and clinically healthy animals (n = 6). The geographical and species-specific distribution of these diagnostic materials is shown in Figure 1.

### 2.2. Ethical Considerations

This study was conducted with the approval of the Ethics Committee of Ankara University (Decision No: 2024-04-24).

### 2.3. Nucleic Acid Extraction and RT-PCR-Based Screening and Genome Determination Reactions

Viral RNA was extracted from 1:10 (*w*/*v*) fecal suspensions using the Biospin Virus DNA/RNA Extraction kit (BioFlux, Bioer, Hangzhou, China), in accordance with the manufacturer’s protocol. All RNA extracts were stored at −80 °C until analysis. Reverse transcription was carried out using the RevertAid First Strand cDNA Synthesis Kit (Thermo Fisher Scientific, Waltham, MA, USA), following the manufacturer’s instructions.

All fecal samples were initially screened by RT-PCR targeting the 3D^(RdRp)^ gene region of bopiviruses, following the screening primers and protocol of László et al. [3], using DreamTaq DNA polymerase (Thermo Fisher Scientific) according to the manufacturer’s recommendations (Table S1). Samples that tested positive were subsequently subjected to VP1-specific RT-PCR using a bopivirus-specific VP1 primer pair designed for all known bopivirus sequences [3] (Table S1). For the VP1-positive sample, the complete coding sequence was determined by combining multiple RT-PCR reactions with overlapping products and a primer-walking strategy (Table S1). The 3′ end of the genome was determined using the 3′/5′ RACE (rapid amplification of cDNA ends) Kit, 2nd Generation (Roche Diagnostics, Mannheim, Germany). All primers used in this study, along with their genomic positions and expected amplicon sizes, are listed in Table S1. PCR products were sequenced by the Dye-terminator (Sanger) sequencing technique through a commercial service provided by Ankara University Rare Diseases Application and Research Center (Ankara, Türkiye). The complete coding sequence of Bopivirus/Sheep/KS-1M/2024/TUR and partial 3D^(RdRp)^ sequence of Bopivirus/Sheep/ANK-K30/2017/TUR strains can be accessed in GenBank under accession numbers of PX610292 and PX610293.

### 2.4. Sequence and Phylogenetic Analyses

The generated sequence data were analyzed using the BLASTn service provided by the National Center for Biotechnology Information (NCBI). Sequences were aligned using AliView [10] and the MUSCLE algorithm [11]. Multiple sequence alignments were conducted with all available bopivirus sequences obtained from GenBank.

Phylogenetic analyses were performed using the Maximum Likelihood method with either MEGA X software [12] for partial 3D^(RdRp)^ and complete P1, 2C and 3CD or IqTree [13] for VP1 sequences. The ‘Find Best Model’ tool was used to select the optimal evolutionary models, which were specified in the figure legends of the corresponding phylogenetic trees. All trees were generated with 1000 bootstrap replicates.

Nucleotide (nt) and amino acid (aa) identity comparisons were performed using the Sequence Identity and Similarity (SIAS) online tool (http://imed.med.ucm.es/Tools/sias.html; accession date: 13 November 2025). Pairwise distance (P-dist) matrix was calculated from the complete bopivirus VP1 nt alignment by MEGAX [12], and a frequency distribution histogram was created using Excel 2510 ver.16.0.19 of Microsoft 365. Recombination Detection Program (RDP) ver. 4.101 was applied for distance plot calculations using the Similarities model with a window size of 200 nt and a step size of 20 nt [14]. The potential secondary RNA structure of the 5′ UTR was generated by the Mfold software [15].

## 3. Results

### 3.1. Detection of Bopivirus Based on the 3D^(RdRp)^ Region

Among the 214 diagnostic samples analyzed in this study, only two of the 82 sheep tested positive, corresponding to an overall positivity rate of 2.4% among sheep. The prevalence rate on the farm where Bopivirus/Sheep/KS-1M/2024/TUR was detected was 20% (1/5), whereas Sheep/ANK-K30/2017/TUR originated from a farm from which only a single sample had been collected. Although samples were collected from both diarrheic and clinically healthy sheep, bopivirus was detected exclusively in diarrheic lambs (2/63, 3.1%). The 587 bp-long fragments of the 3D^(RdRp)^ share 97.44% nucleotide (nt) and 98.97% amino acid (aa) pairwise sequence identity. Additionally, based on BLASTn-based sequence analysis, they exhibited at least 95.22% nt and 96.41% aa identity with Bopivirus B viruses, with the highest percentage (≈98%) to an unpublished Bopivirus sp. isolate caprine/China/SWUN/B5 (OP272478) from China. In contrast, their sequence identities with the members of other Bopivirus species (*Bopivirus abovi* and “Bopivirus C”) were significantly lower, with a maximum of 71.03% nt and 72.82% aa identity. Based on sample data, which includes host species, origin provinces, sample code, and year of collection, the viruses identified in this study were named as Bopivirus/Sheep/ANK-K30/2017/TUR (PX610293) and Bopivirus/Sheep/KS-1M/2024/TUR (PX610292).

**Figure 2:**
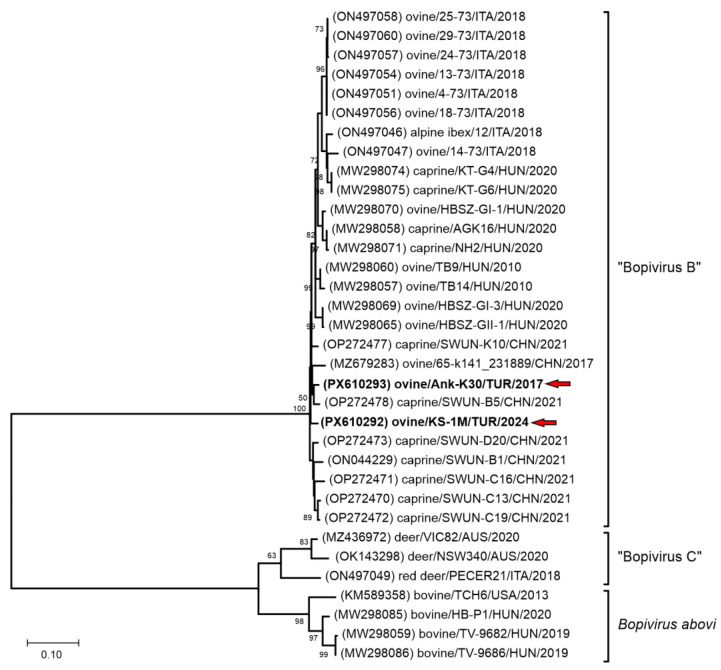
The phylogenetic tree of 587 nt-long partial 3D^(RdRp)^ sequences of all the known bopivirus nucleotide sequences. The Maximum Likelihood phylogenetic tree was constructed with the Tamura-Nei (TN93) + G model with 1000 bootstrap replicates. Only BS values ≥ 50 were present. The study strains were written in bold and marked with red arrows.

Phylogenetic analysis of the partial 3D^(RdRp)^ sequences indicates that the detected viruses clustered within the Bopivirus B lineage with bopiviruses from small ruminants (Figure 2), thereby supporting their classification within this recently described species (“Bopivirus B”).

### 3.2. Complete Coding Sequence Analyses of Bopiviruses Detected

For further molecular characterization and genotype identification of the bopiviruses detected in this study—Bopivirus/Sheep/ANK-K30/2017/TUR and Bopivirus/Sheep/KS-1M/2024/TUR—multiple RT-PCR reactions with bopivirus-specific primer pairs (Table S1) generating overlapping products were performed. The generated PCR products were sequenced by Sanger sequencing with the primer walking method. With this technique, the 7303 nt-long (the c.a. 68 nt-long 5′ end is missing) complete coding sequence (CDS) of Bopivirus/Sheep/KS-1M/2024/TUR could be determined. Unfortunately, only a 359 nt- and a 710 nt-long partial VP3 and partial 2A-2C were successfully obtained from Bopivirus/Sheep/ANK-K30/2017/TUR which show 86.62% nt/92.43% aa and 94.67% nt/97.04% aa identity to the corresponding genome parts of Bopivirus/Sheep/KS-1M/2024/TUR, respectively.

The determined CDS of Bopivirus/Sheep/KS-1M/2024/TUR was predicted to contain a single open reading frame (ORF) and follows the general genome layout of bopiviruses: 5′UTR-ORF: [VP4-VP2-VP3-VP1-2A-2B-2C-3A-3B-3C-3D]-3′UTR.

The 620 nt-long partial 5′UTR shows 97.27% nt identity to the most identical 5′UTR of Bopivirus sp. strain ovine/TB14/2010-HUN (MW298057) and shows similar secondary RNA structure as the type II IRES of bopiviruses. The 77 nt-long 3′UTR also shows the highest (97.4%) sequence identity to the corresponding genome part of ovine/TB14/2010-HUN.

**Figure 3:**
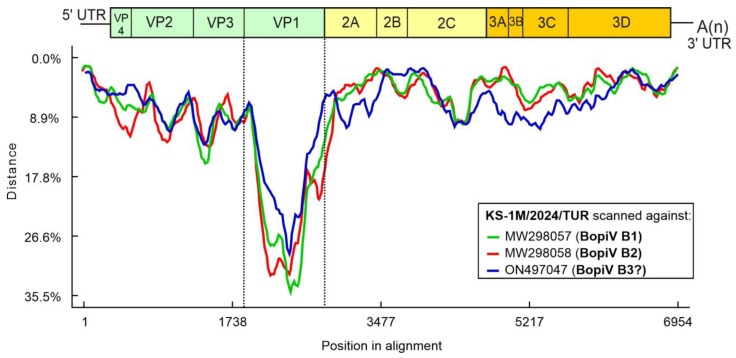
Distance plot of representative genotypes of species “Bopivirus B” (for legends see the insert of the figure) compared to the complete coding sequence of study strain KS-1M/2024/TUR as a query in a multiple nt alignment with a window size of 200 bp and a step size of 20 bp. Vertical dotted lines indicate the borders of VP1.

The 6606 nt-long ORF encodes a 2201 aa-long single viral polyprotein which shows 91.89% nt and 95.32% aa pairwise identity to the closest match of Bopivirus/sheep/14-73/2018/ITA (ON497047) of species “Bopivirus B” identified by BLAST searches. Nucleotide distance plot of the complete CDS of study strain Bopivirus/Sheep/KS-1M/2024/TUR scanned against the representative members of species “Bopivirus B” including Bopivirus/sheep/14-73/2018/ITA show the relatively small sequence divergence ranged between 1.3% (at 5′UTR) and 15.8% (at VP3) across the CDS of the study strain except the VP1 region where the divergence is up to 35.5% indicates that VP1 shows the highest divergence relative to other regions (Figure 3).

**Figure 4:**
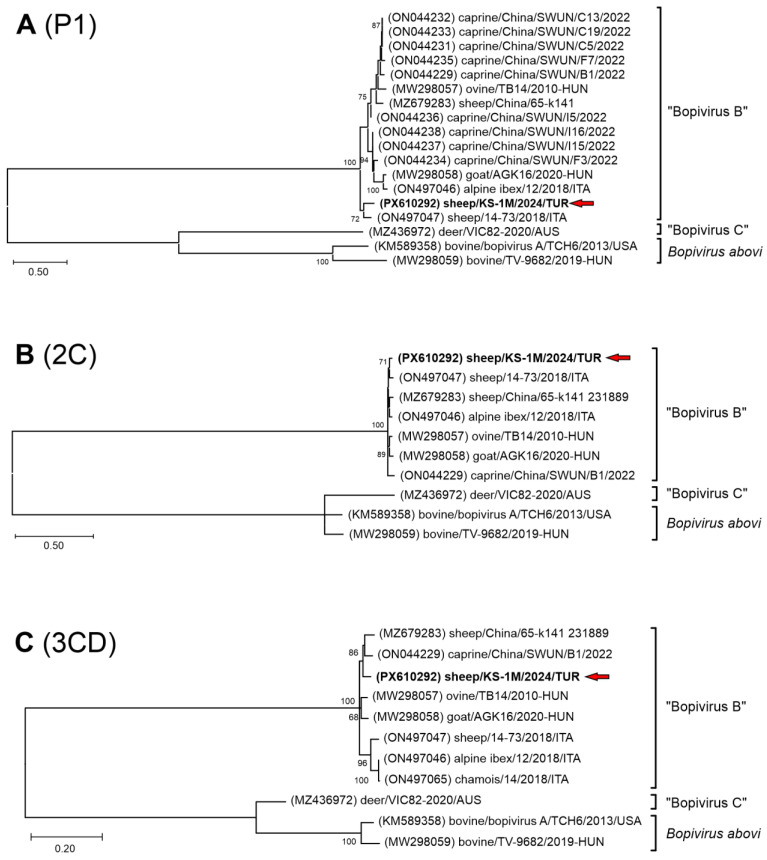
The phylogenetic trees of complete P1 (**A**), 2C (**B**) and 3CD (**C**) nucleotide sequences of the study strain Bopivirus/sheep/KS-1M/2024/TUR and all the known bopiviruses. The Maximum Likelihood phylogenetic trees were constructed with the General Time Reversible (GTR) + G + I (**A**), Kimura-2 (K2) + G + I (**B**) and Tamura-Nei (TN93) + G + I (**C**) models with 1000 bootstrap (BS) replicates. Only BS values ≥ 50 were present. The study strain was written in bold and marked with a red arrow.

**Figure 5:**
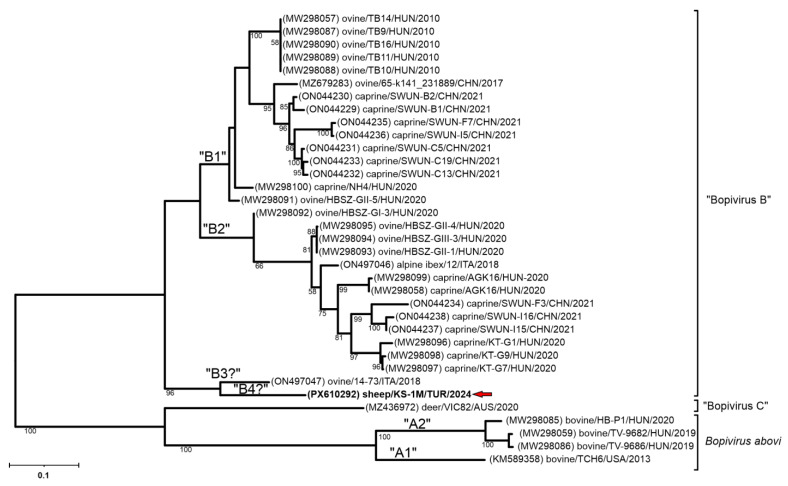
The phylogenetic tree of VP1 nucleotide sequences of the study strain Bopivirus/sheep/KS-1M/2024/TUR and all the known bopiviruses. The Maximum Likelihood phylogenetic tree was constructed with the General Time Reversible (GTR) + G model with 1000 bootstrap (BS) replicates. Only BS values ≥ 50 were present. The study strain was written in bold and marked with a red arrow.

The nt phylogenetic trees of complete 2C and 3CD non-structural regions also indicate a close relationship of the study Bopivirus/Sheep/KS-1M/2024/TUR strain to all the members of species “Bopivirus B” (Figure 4B,C), while in the P1 and VP1 capsid trees Bopivirus/Sheep/KS-1M/2024/TUR is located on a distinct lineage together with Bopivirus/sheep/14-73/2018/ITA among “Bopivirus B” viruses but also considerably separated from each other (Figure 4A and Figure 5).

In the VP1 genomic region, Bopivirus/Sheep/KS-1M/2024/TUR also showed the highest sequence identity to Bopivirus strain sheep/14-73/2018/ITA (ON497047), with 82.51% nt and 86.31% aa identity. In contrast, its sequence identities with the other “Bopivirus B” strains are less than 76.87% and even lower (≤52.11%) with the members of *Bopivirus abovi* and “Bopivirus C” (Table 1).

**Figure 6:**
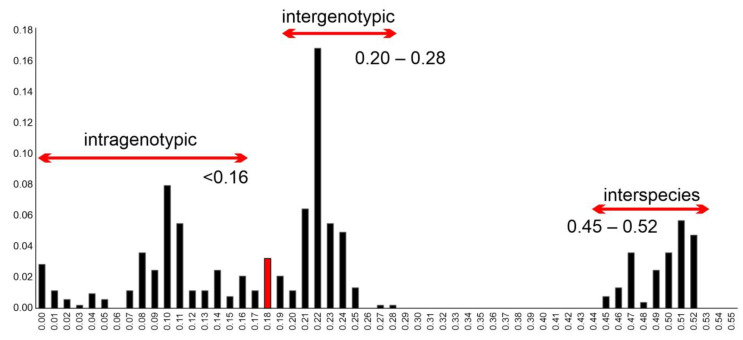
Frequency distribution of pairwise distances (p-distances) between the n = 33 VP1 nucleotide sequences including the study strain Bopivirus/Sheep/KS-1M/2024/TUR and all known bopivirus VP1 sequences with possible intra-, intergenotypic and interspecies distance ranges proposed earlier [3]. Red bar indicates the lowest p-distance (0.18) between the study strain and the most closely related bopivirus strain sheep/14-73/2018/ITA (ON497047).

In the frequency distribution of pairwise VP1 nt distances, the lowest calculated p-distance of Bopivirus/Sheep/KS-1M/2024/TUR was 0.18 compared to the most closely related bopivirus strain sheep/14-73/2018/ITA (ON497047), which is higher than the intra-, but lower than the intergenotypic distance ranges proposed by Laszló et al. [3] (Figure 6). All other p-distances of Bopivirus/Sheep/KS-1M/2024/TUR are ≥0.23 which is in the range of intergenotypic p-distances. The p-distances of sheep/14-73/2018/ITA (≥0.21) are also within this range.

**Figure 7:**
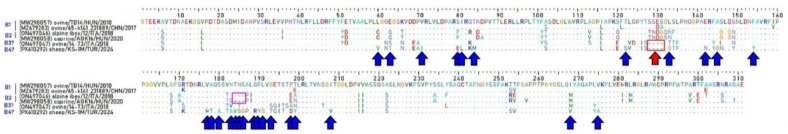
Amino acid alignment of the full-length VP1 sequences of Bopivirus/Sheep/KS-1M/2024/TUR and selected representatives of “Bopivirus B” genotypes. A red arrow and a red box indicate a position of a deletion between S127 and G132 common in Bopivirus/Sheep/KS-1M/2024/TUR and sheep/14-73/2018/ITA. Blue arrows indicate the unique aa substitutions of Bopivirus/Sheep/KS-1M/2024/TUR. A magenta box indicates the position of a unique 3-aa-long deletion of “B2” strains.

Meanwhile, comparison of the full-length Bopivirus/Sheep/KS-1M/2024/TUR VP1 aa sequence to selected representatives of “Bopivirus B” genotypes, including the strain sheep/14-73/2018/ITA (ON497047), revealed various amino acid deletions and substitutions that set Sheep/KS-1M/2024/TUR and sheep/14-73/2018/ITA apart from genotypes of “B1” and “B2” like a four–amino acid deletion between S127 and G132 compared to strains of genotypes “B1” and “B2” (Figure 7). There are also aa substitutions which also make Sheep/KS-1M/2024/TUR different from sheep/14-73/2018/ITA (Figure 7). Interestingly the four–amino acid deletion between N184 and A188 previously reported in B2 genotype strains [7] is not observably in the corresponding genome region of the study strain.

## 4. Discussion

Although many *Picornaviridae* viruses are well characterized, several newly identified members—such as hunnivirus, kobuvirus, boosepivirus, and bopivirus—have been increasingly reported in recent years [3,5,6,7,8,9,16,17,18]. Since the detection of the first bopivirus genome (Bopivirus A1; KM589358) in 2013, bopiviruses have been identified in a wide range of domestic and wild animal species in numerous countries [3,4,5,6,7,8,9]. Collectively, current evidence indicates that bovine strains belong to *Bopivirus abovi*, sheep and goat strains to “Bopivirus B”, and deer strains to “Bopivirus C”, highlighting the increasing diversity and host range within this emerging group of picornaviruses. Despite their widespread occurrence, there remains a need for comprehensive studies to elucidate their epidemiological patterns, the factors influencing their transmission, the genetic characteristics of these viruses and, their potential role as causative agents of diarrhea.

Studies on calf diarrhea in Türkiye have predominantly focused on rotaviruses and coronaviruses, reporting both their epidemiological roles and molecular characteristics [19,20,21]. Additional research has documented several other viral agents—including caliciviruses, picornaviruses, and astroviruses—in diarrheic calves [22,23,24,25]. In contrast, investigations targeting viral enteropathogens in sheep and goats remain scarce, with only a few reports available [16,26,27].

This study aimed to investigate the prevalence and genotype diversity of bopiviruses in sheep, goats, and cattle across a broad geographical range in Türkiye over an extended period (2009–2024), including retrospective fecal samples from numerous farms (Figure 1). Among the sheep samples analyzed, two (2.4%; 2/82) tested positive for bopivirus, thus providing the first evidence of bopivirus presence in Turkish sheep. However, the overall positivity rate was lower than reported in Italy (10.9%; 14/128) [5] and Hungary (36.2%; 17/47) [3], but the farm-level prevalence (20% in the farm of Bopivirus/Sheep/KS-1M/2024/TUR) is in the reported range. Variations in epidemiological data across countries are expected due to differences in husbandry practices, sampling criteria, and clinical status. Among the samples analyzed in the present study, none of the cattle and goat fecal samples tested positive by RT-PCR. Although these species were sampled in the same provinces as the positive sheep, limited information on sampling years, farm locations, and grazing practices prevents any inference about cross-species transmission. More extensive studies are required to better understand the epidemiology of bopivirus across different host species in Türkiye.

Bopivirus has been detected in both clinically healthy and diarrheic animals, with higher prevalence in young ruminants and lower apparent epidemiological significance in adults [3,5,7]. Notably, co-infections with other picornaviruses—such as caprine enterovirus, kobuvirus, and hunnivirus—have also been reported [7]. Overall, information on the biological significance and true prevalence of bopivirus remains limited. In the present study, bopivirus was found exclusively in samples from sheep suffering from diarrhea (2/63, 3.1%), and the two bopiviruses identified were detected in diarrheic lambs. Although the detection of positive cases exclusively in diarrheic lambs may suggest an age-related pattern, the low prevalence prevents any definitive conclusions. It should also be noted that the sample, in which Bopivirus/Sheep/ANK-K30/2017/TUR was identified, had been included in a previous study [26] and tested for additional viral pathogens. That sample was found to be positive for rotavirus and picobirnavirus, whereas the Bopivirus/Sheep/KS-1M/2024/TUR sample tested negative for rotavirus in a routine diagnostic screening.

Gastroenteritis severity reflects the interplay between the types of co-infecting agents, viral load and host immunity. High viral replication promotes epithelial damage and barrier dysfunction, while effective immune responses limit replication but may drive symptoms via inflammation. Based on current knowledge bopiviruses are widespread in ruminant species, as more data become available, the potential contribution of bopivirus—either alone or in mixed infections—to diarrheal disease will likely become clearer.

The 3D^(RdRp)^ gene of picornavirus is relatively conserved, yet it also exhibits genetic diversity associated with geographic and host factors. These features make the 3D^(RdRp)^ gene a suitable target for molecular epidemiological analyses. Phylogenetic analyses based on the 3D^(RdRp)^ gene region (Figure 2), together with identity data —showing at least 95.22% nt and 96.41% aa identity with “Bopivirus B” viruses, whereas identities with other bopiviruses (*Bopivirus abovi* and “Bopivirus C”) were at most 71.03% nt and 72.82% aa identity—indicate host-specific clustering of bopivirus genotypes [3,5,6,7]. Consistent with these observations, the viruses detected in sheep in this study, Bopivirus/Sheep/ANK-K30/2017/TUR and Bopivirus/Sheep/KS-1M/2024/TUR, clustered within “Bopivirus B” (Figure 2).

Epidemiological investigations of circulating bopivirus genotypes will enhance current knowledge and inform assessments of interspecies transmission, host range restrictions, and clinical impacts, especially among small ruminants. László et al. [3] described two VP1 genotypes (B1 and B2) circulating among sheep and goats in Hungary, regardless of host species. The complete VP1 sequence obtained in this study is 921 bp long, slightly shorter than the B2 (924 bp) and B1 (933 bp) genotypes registered in GenBank. Yang et al. [7] reported a three-amino acid deletion between N184 and A188 in all Bopivirus B2 strains compared to the Bopivirus B1 genotypes. Our analysis indicate that this deletion is present exclusively in B2 genotype bopiviruses within Bopivirus B. Interestingly, both the Bopivirus/Sheep/KS-1M/2024/TUR and the Italian sheep/14-73/2018/ITA (ON497047) strains harbor a four–amino acid deletion between positions S127 and G132, distinguishing them from the “B1” and “B2” genotypes; however, the Bopivirus/Sheep/KS-1M/2024/TUR strain also contains multiple additional unique amino acid substitutions. Nucleotide and amino acid identity analyses (Table 1) show 82.51% and 86.31% identity between these two strains, respectively, while identity with other Bopivirus B genotypes does not exceed 76.87% nt and 82.08% aa. Bopivirus/Sheep/KS-1M/2024/TUR is most closely related to a sheep Bopivirus B strain (sheep/14-73/2018/ITA) from Italy (ON497047), but the phylogenetic separation, the low sequence identities and high p-distance values and several unique aa mutations in VP1 compared to existing genotypes of “B1” and “B2” suggest that both strains could belong to either a single (“B3”) or two novel genotypes (“B3” and “B4”) in species “Bopivirus B”, although additional closely related sequences are necessary for proper typing.

In conclusion, this study demonstrated the presence of Bopivirus B in sheep in Türkiye and provided preliminary insights into its possible epidemiological characteristics. The findings also suggest two novel Bopivirus B genotypes, underscoring the VP1 genetic diversity observed in both the Bopivirus/Sheep/KS-1M/2024/TUR and a Bopivirus/sheep/14-73/2018/ITA (ON497047) from Italy. Further studies are warranted to elucidate the pathogenic potential and clinical significance of Bopivirus B in sheep and other ruminant species. Continued surveillance efforts, together with comprehensive molecular characterization and epidemiological investigations, will contribute to a better understanding of Bopivirus species and its implications for animal health.

## Figures and Tables

**Table 1:** Nucleotide (%) and amino acid (%) identities of VP1 gene sequences between Bopivirus/Sheep/KS-1M/2024/TUR and Bopivirus sequences deposited in GenBank. The highest identity values are marked with bold.

Species	Genotype	Acc No.	Nucleotide (%)	Amino Acid (%)
			KS-1M	KS-1M
“Bopivirus B”	B3?	ON497047	**82.51**	**86.31**
	B2	MW298093	73.54	77.3
		MW298094	73.6	77.51
		MW298095	73.48	77.51
		ON497046	74.7	78.17
		MW298058	75.57	78.17
		MW298099	75.13	77.19
		MW298096	73.61	75.57
		MW298097	75.02	77.85
		MW298098	74.59	77.52
		ON044234	74.59	79.47
		ON044237	74.48	78.82
		ON044238	74.15	78.17
		MW298092	73.51	77.96
	B1	MW298091	74.08	79.06
		MZ679283	76.43	81.75
		ON044229	76.76	81.75
		MW298057	76.76	81.43
		MW298088	75.44	79.47
		MW298087	75.44	79.47
		MW298089	75.44	79.47
		MW298090	75.44	79.47
		ON044236	76.76	81.1
		ON044235	76.33	80.78
		ON044231	76.87	82.08
		ON044232	76.65	81.75
		ON044233	76.22	80.45
		ON044230	76.65	82.08
*Bopivirus abovi*	A1	KM589358	50.81	47.23
	A2	MW298059	49.83	43.64
“Bopivirus C”	C1	MZ436972	52.11	49.51

## Data Availability

Data available in a publicly accessible repository: The data presented in this study are openly available in GenBank database under the accession numbers PX610292 and PX610293.

## Supplementary Materials

The following supporting information can be downloaded at: https://www.mdpi.com/article/10.3390/pathogens15010052/s1, Table S1: List of the primers used for screening, typing and primer-walking reactions of this study.
