# Supplementary material for: Potential Novel Genotype of “Bopivirus B” from Sheep in Türkiye: Epidemiology and Molecular Characterization
Source: Pathogens. 2026 Jan 5;15(1):52. doi: 10.3390/pathogens15010052 (PMC12845433; doi:10.3390/pathogens15010052)
Supplement: Supplementary file 1 [file pathogens-15-00052-s001.zip › pathogens-4056675-supplementary.pdf]

Table S1. List of the primers used for screening, typing and primer-walking reactions of this study.

**Name**	**Sequence (5′-3′)**	**Annealing temperature (°C)**	**Estimated PCR product length (bp)**	**References**
HBG-3D-Screen-F	CTGRGCAAGTTCACCAACAA	55	627	László et al. (2021)
HBG-3D-Screen-R	GTCCATGACAGGGTGAATCA			
Ovipi-VP1-Fgen	TCTGCAACCGACTWCCGCTA	60	1169	
Ovipi-VP1-Rgen	TTGGWYTCAATGTCACCACC			
BopiV-158-F	TCTCCGAAGCTTGCATGATTCT	58	1100	In this study
BopiV-1100-R	GGTTTCCAAATCGGCAAGCA			
BopiV-866-F	CAACACTACCTCTGGTAACTCTGG	62	1000	
BopiV-1862-R	GGTCTGGTCGGTGGGTTCC			
BopiV-1791-F	GTTGGCGAAGGCATGGTGG	60	796	
BopiV-2587-R	GGGACGACTTCAAGTCGGGA			
BopiV-3345-F	TGCCCCAGACCTTTCTGGAC	60	730	
BopiV-4075-R	CAGTGGGCGTTCTTTGCCA			
BopiV-4007-F	CCTAGAATACACTCCCGAGGAC	58	850	
BopiV-4856-R	GCCTTGGGATCTTGCGAAC			
BopiV-4795-F	GCTTTCGTAACGATTGCCCATT	60	1040	
BopiV-5837-R	CTTAGCAACCACCGGCGAA			
BopiV-5739-F	GAGACGGTTCCCATAATCGG	60	1026	
BopiV-6765-R	CTTTGTAGTCAAAATCATACACGT			
BopiV-7140-F	GACAAGAGTGGGGTATTTAACC	61	400	
OligodT-Anchor-Adapter	GACCACGCGTATCGATGTCGACTTTTTTTTTTTTTTTTV			5'/3' RACE kit (Roche Diagnostics, Mannheim, Germany)
Adapter	GACCACGCGTATCGATGTCGAC			
